# Using RNase sequence specificity to refine the identification of RNA-protein binding regions

**DOI:** 10.1186/1471-2164-9-S1-S17

**Published:** 2008-03-20

**Authors:** Xin Wang, Guohua Wang, Changyu Shen, Lang Li, Xinguo Wang, Sean D Mooney, Howard J Edenberg, Jeremy R Sanford, Yunlong Liu

**Affiliations:** 1 Division of Biostatistics Department of Medicine, Indiana University School of Medicine, Indianapolis, IN 46202, USA; 2 Center for Computational Biology and Bioinformatics, Indiana University School of Medicine, Indianapolis, IN 46202, USA; 3 College of Automation, Harbin Engineering University, Harbin, Heilongjiang 150001, China; 4 School of Computer Science and Technology, Harbin Institute of Technology, Harbin, Heilongjiang, 150001, China; 5 The Center for Genomics and Bioinformatics, Indiana University, Bloomington, IN 47405, USA; 6 Department of Medical and Molecular Genetics, Indiana University School of Medicine, Indianapolis, IN 46202, USA; 7 Department of Biochemistry and Molecular Biology, Indiana University School of Medicine, Indianapolis, IN 46202, USA; 8 Center for Medical Genomics, Indiana University School of Medicine, Indianapolis, IN 46202, USA

## Abstract

Massively parallel pyrosequencing is a high-throughput technology that can sequence hundreds of thousands of DNA/RNA fragments in a single experiment. Combining it with immunoprecipitation-based biochemical assays, such as cross-linking immunoprecipitation (CLIP), provides a genome-wide method to detect the sites at which proteins bind DNA or RNA. In a CLIP-pyrosequencing experiment, the resolutions of the detected protein binding regions are partially determined by the length of the detected RNA fragments (CLIP amplicons) after trimming by RNase digestion. The lengths of these fragments usually range from 50-70 nucleotides. Many genomic regions are marked by multiple RNA fragments. In this paper, we report an empirical approach to refine the localization of protein binding regions by using the distribution pattern of the detected RNA fragments and the sequence specificity of RNase digestion. We present two regions to which multiple amplicons map as examples to demonstrate this approach.

## Introduction

RNA-protein interactions influence virtually every step of post-transcriptional gene expression including pre-mRNA capping, splicing, polyadenylation and mRNA export, stability, localization and translation [1]. These reactions are not only essential steps in the expression of most eukaryotic genes, but also provide key points for the regulation of gene expression and may even increase the proteomic complexity encoded by a genome [2]. The human genome encodes at least 673 genes with predicted RNA binding activity (GO:00003723, ). Mutations disrupting function of either *cis*-acting RNA elements or *trans*-acting RNA binding proteins (RBP) form the basis for many heritable human diseases [3-5]. Despite the profound impact of RNA binding proteins on cellular metabolism, we still know little regarding the RNA binding specificity of these important factors.

The analysis of RNA binding proteins is further complicated by the observation that many are multifunctional [1]. Elucidating the functions of RNA binding proteins require detailed biochemical analyses, and are constantly challenged as novel biological roles for these proteins are described; this process is usually slow. A more effective method to understand the roles of RNA binding proteins in RNA processing is to allow the specificity of RNA-protein interactions to establish connections with mRNA or ncRNA (non-coding RNA) metabolism. Thus truly unbiased, genome-wide methods capable of preserving the *in situ* RNA binding specificity are required to elucidate the functions of RNA binding proteins in an efficient and comprehensive manner.

The most widely used technique for studying the interactions of RNA binding proteins with their RNA targets *in vivo* may be the immunoprecipitation (IP)-microarray assay [4,5]. RNA binding proteins are immunoprecipitated from cultured cells or tissues and co-purifying RNA is used to probe expression arrays. However, under these conditions RNA binding proteins are free to dissociate from their endogenous RNA targets and re-associate with higher affinity binding sites, thus there is great risk of false discovery [6]. The cross-linking immunoprecipitaion (CLIP) assay can overcome these drawbacks while facilitating identification of RNA targets [7,8]. CLIP analysis has been successfully used to study the murine RNA binding protein NOVA, and also reveal the specific binding sites within the captured pre-mRNAs [7,8].

In order to conduct global profiling of the RNA sites bound by RNA binding proteins *in vivo*, we have coupled the CLIP purification strategy from with high-throughput pyrosequencing of CLIP amplicons; we call this pyro-CLIP technology (pCLIP). The 454 sequencing platform (454 Life Sciences, Branford, CT) is an integrated system of emulsion-based PCR amplification of hundreds of thousands of DNA/RNA fragments linked to high throughput parallel pyrosequencing in picoliter-sized wells [9-11]. Pyrosequencing allows for comprehensive sequencing of the CLIP amplicon pool and is extremely cost-effective relative to Sanger sequencing.

Pyro-CLIP experiments generate hundreds of thousands of RNA fragments (CLIP amplicons) to which an RBP binds. Substantial bioinformatics analysis is required to accurately identify the protein binding regions genome-wide. When the experimentally-detected RNA fragments (CLIP amplicons) are aligned to the human genome, many regions are observed to be encompassed by multiple fragments. In this study, we developed an empirical approach to refine the detection of protein binding regions by using the distribution pattern of the detected RNA fragments and the sequence specificity of RNase digestion along the amplicon-enriched genomic region.

## Methods

### Data Structure

**Figure 1:**
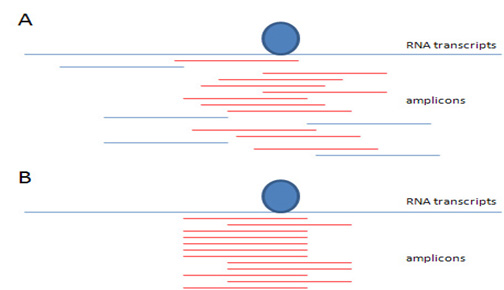
**Distribution of detected amplicons in the pyro-CLIP experiment.** A. Detected and undetected sequence fragments. B. Distribution of detected amplicons in the experiment.

Pyro-CLIP experiments result in hundreds of thousands of amplicons (RNA sequences with 50 to 70-bp in length). Preliminary data suggested that in a pyro-CLIP experiment, dozens of amplicons were usually detected within one genomic region. Hypothetically, all the detected amplicons should encompass at least one *cis*-acting element recognized by the studied RNA binding protein. The number of detected amplicons in each genomic region depends on various factors including the abundances of RNA transcript. Fig. 1A depicts a schematic view of this problem. Intuitively, all the red amplicons will be detected in the pyro-CLIP experiment since their sequences encompass the protein binding sites. On the contrary, no blue sequences are visible because they were not cross-linked to the RNA-protein complex, and therefore will not be immunoprecipitated by the antibody.

In the preliminary data, we found that the distribution of detected amplicons in each genomic region is similar to the pattern shown in Fig. 1B. This clearly indicates that the partial RNase digestion trimmed RNA fragments in a sequence-specific manner [12]. This feature allows us to refine the detection of protein binding regions from pyro-sequencing-derived RNA fragments. In this study, we propose a computational approach to achieve this goal by using two types of information, the detected pattern of RNA amplicons (Fig. 1B), and the estimated specificity of RNase digestion along the genomic sequence.

The overall procedure includes three key steps: (1) pre-processing of CLIP amplicons, (2) estimation of sequence specificity of RNase digestion, and (3) prediction of RNA binding regions.

### Pre-processing of CLIP amplicons

In the pyro-CLIP experiment, two short RNA linker sequences were ligated on both 5′- and 3′-end of each amplicon. These linker sequences were used as nested primer binding sites for reverse transcription and amplification. After removing both 5′- and 3′-end linker sequences, the remaining amplicons were mapped back into human genome using *blastn* program [13]. In the remaining analysis, we only focus on the genomic regions that contain multiple amplicons.

In practice, the number of genomic regions that match with the query amplicon varies depending on the selection of parameters while using the *blastn* program [13]. We removed all the genomic regions whose length fell out of 50 to 70-bp range. We also removed the amplicons that match more than 20 different genomic locations, which are from repetitive sequences and can't be mapped uniquely.

### Estimation of the sequence specificity of RNase digestion

In the pyro-CLIP assay, RNase digestion was used to trim the cross-linked RNA fragments to 50-70 nucleotides. Instead of cutting RNA fragments at random locations, RNase digestion has sequence specificity. The RNases used in the experiment were RNase I and RNase T1 (Applied Biosystems, Foster City, CA).

After mapping the detected amplicons back to the genome, we observed a large number of RNA fragments that shared identical 5′- and 3′-end genomic locations as in Fig 1B, rather than overlapping a site with in a ragged pattern as in Fig. 1A. This is a strong indication that RNase prefers to digest RNA sequences at some specific sequences. In this study, we estimated sequence specificities of RNase digestion on all possible 6-bp motifs.

**Figure 2:**
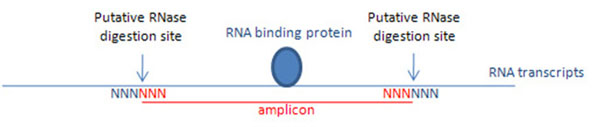
Estimation of the sequence specificity of RNase digestion.

Each detected amplicon was generated by two RNase digestion sites (Fig. 2). After mapping back into the human genome, the two RNase digestion sites on each amplicon can be easily determined by combining 3-bp sequences on both edges of the amplicon (red Ns in Fig. 2), and their natural extensions in the local genomic region (blue Ns in Fig. 2). We calculated the number of cutting events on each of 4^6^=4096 potential 6-bp motifs based on all the detected amplicons and their mapped genomic locations.

The distribution of 6 nt sequences in the human transcriptome is not random, so in order to estimate sequence specificity of RNase digestion, the number of cutting events on each 6-bp motif should be corrected based on the frequency of the site. For instance, all the motifs that contain a CpG di-nucleotide appear in a much lower frequency than the ones without. Therefore, a lower number of detected cutting events on these motifs do not necessarily imply a low sequence specificity of RNase digestion. Based on this principle, we estimated the likelihood of RNase digestion on each 6-bp motif by normalizing the number of detected cutting events to the frequency of occurrence of the motif in the human transcriptome:

$$L_{k}=\frac{f_{k}/f_{0k}}{\sum_{i=1}^{N}f_{i}/f_{0i}}$$

where *L_k_* depicts the likelihood of RNase digestion on the *k*-th 6-bp motif; *f_i_* denotes the number of cutting events detected on the *i*-th 6-bp motif; and *f_0i_* represents its frequency of occurrences in the human transcriptome (*N=*4096 is the total number of potential 6-bp motifs).

### Refining the detection of protein binding regions

**Figure 3:**
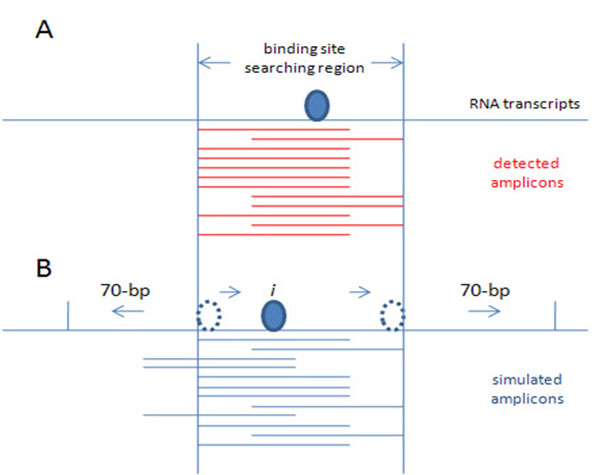
**Searching strategy**. A. Distribution of detected amplicons in the experiment. B. Distribution of detected amplicons in the simulation.

The size of detected amplicons ranges from 50 to 70 nucleotides. If there were no sequence specificity to the RNase digestion, the overlapped pattern of multiple amplicons would provide very good resolution of the detection of protein binding (Fig. 1A). However, in many circumstances, the pattern of amplicons we observed is more similar to Fig. 1B, or even Fig. 3C, suggesting bias due to sequence specificity of RNase digestion. We report an empirical approach to improve the resolution of binding regions using the likelihood of RNase digestion (estimated in Eq. 1) within local genomic regions.

Our strategy is based on several assumptions:

1) All the detected amplicons should encompass the *cis*-acting element to which the protein binds;

2) The probability of each being selected as an RNase digestion site is proportional to the estimated likelihood of RNase digestion on its 6-bp motif, calculated in Eq. 1;

3) Each genomic region where multiple amplicons are detected is located within one RNA transcript;

For each genomic region in which multiple amplicons are detected, we simulate the distribution of amplicons that would occur based upon RNase sequence specificity, and then simulate the subset that would be selected if a protein bound to a specific site. This simulation is repeated as the binding site is moved along the RNA. We then compare the distribution of experimentally-detected amplicons (Fig. 3A) and of simulated amplicons (Fig. 3B); the genomic region resulting with highest similarity was regarded as the protein binding region. In brief, the proposed empirical approach includes the following steps:

1) Randomly generate a large number of RNA fragments based on the estimated likelihood of RNase digestion (Eq.1) along the genomic region in which multiple amplicons were detected plus a 70-bp extension towards both 5′- and 3′-side (Fig. 3B).

2) For each possible protein binding site (*i*), randomly select *M* simulated amplicons from among the RNA fragments generated in the previous step that encompass the current site (*i*). *M* is the number of experimentally-detected amplicons (Fig. 3A).

3) For each genomic locus *j* within the searching region, calculate the number of encompassing amplicons assuming protein binding occurs at the *i*-th locus, defined as F(*i*,*j*), in both experimentally-determined group and simulated group; for each group, **F(*i*)** is a vector whose number of elements is equal to the length of genomic region being searched.

4) Calculate the *Pearson* correlation coefficient of the resultant **F(*i*)** vector between experimentally-detected amplicons and simulated amplicons assuming that the protein binds on the *i*-th genomic locus, C(*i*).

5) Continue steps 2 to 4 for the entire region. The region that results in highest correlation coefficient will be regarded as the region with the highest binding probability.

### Evaluation of the effects of various biological variations on binding site prediction

In order to evaluate the robustness of the proposed approach to various noises resulted from multiple biological and computational steps, we created a simulated sets of virtual amplicons based on the sequences of a randomly-selected RNA region, and a pre-defined protein binding site. While generating these testing data, we deliberately included noises from various sources, including:

1) Antibody non-specificity;

2) Inaccurate estimation of the likelihood of RNase digestion on different 6-bp motifs;

3) Noises associating with BLAST search.

We tested the performance of the proposed approach under different levels of measurement noise based on the capability of the model to predict the pre-defined protein binding site.

## Results

### Estimation of the sequence specificity on RNase digestion

**Figure 4:**
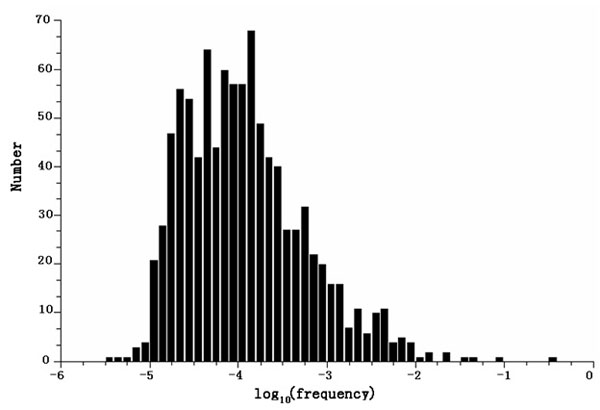
Histogram of the estimated likelihood of RNase digestion on 967 6-bp motifs that occur at least once on the edge of the experimentally detected RNA fragments.

In the preliminary dataset (unpublished data), a total of 29,071 RNA fragments ranging from 50-70 bp in length were used to estimate the sequence specificity of RNase digestion. We calculated the number of occurrences of all the potential 6–bp motifs at both ends of the RNA fragments, totaling 58,142 6-bp elements. We observed significant preference of RNase digestion on some 6-bp motifs. Only 967 of the 4,096 potential 6-bp motifs (24%) were observed as digestion sites. Strikingly, 26% of RNase digestion occurs at AATAAA sites. Fig. 4 demonstrates the histogram of the cutting frequencies on all the 6-bp motifs that have at least one cutting event. The top 1% are listed in Table 1. To quantitatively evaluate the sequence specificity of RNase digestion, we calculated the entropy of RNase cutting frequency at all the potential 6-bp motifs: E=-Σ(p_i_*log*_N_p*_*i*_), where *p_i_* is the probability that 6-bp motif *i* is selected as cutting site *p_i_*=*f_i_*/ Σ*f_i_*. By definition, the resultant entropy should be a number from 0 to 1. A larger entropy implies less sequence specificity of the RNase digestion. The entropy for the frequencies of enzyme digestion on 6-bp motif is 0.42. This suggests that RNase digestion has significant preference for certain motifs. This finding is different from the reported cleavage specificity by the manufacturer, where RNase A cleaves at 3′ of single strand C's and U's, and RNase T1 cleaves at 3′ of single strand G's ().

**Table 1:** Top 1% 6-bp motifs and their RNase digestion frequencies

human transcriptome (entropy=0.96)		detected RNA fragments (entropy=0.42)		adjusted frequencies (entropy=0.56)	
6-bp	frequency	6-bp	frequency	6-bp	frequency
AAAAAA	0.0035	AATAAA	0.262	TCTACA	0.333
TTTTTT	0.0035	TCTACA	0.054	AATAAA	0.085
AAAAAT	0.0016	TTGAAT	0.033	TTGAAT	0.044
ATTTTT	0.0016	AACAGA	0.021	CCTACA	0.036
AAAATA	0.0015	AACAAG	0.019	AACAGA	0.024
TATTTT	0.0013	CCTACA	0.019	AACAAG	0.023
TAAAAA	0.0013	TCTGAA	0.018	TCTGAA	0.016
TTTTTA	0.0013	TTCAGA	0.014	TTCAGA	0.016
AAATAA	0.0013	ATTCTT	0.013	CGTGAA	0.011

In vertebrate genomes, some sequences appear less frequently than others. For example, the number of occurrences of motifs that contain a CpG dinucleotide is significantly reduced. Therefore, we calculated the frequencies of all the 6-bp motifs in human transcriptome, and further estimated the likelihood of RNase digestion on each potential 6-bp motif based on Eq. 1. With this adjustment, the entropy level of cutting likelihood becomes 0.56. Top 1% 6-bp motifs with highest cutting likelihood are listed in Table 1.

### Estimation of binding sites of RNA-binding protein

The 29,071 RNA fragments cover 52 non-overlapping genomic regions that contain at least 10 fragments. Most enriched regions contain one or two protein binding sites. In this report, we use two examples to demonstrate the potential application of the proposed approach.

1) Genomic regions that contain one protein binding site

**Figure 5:**
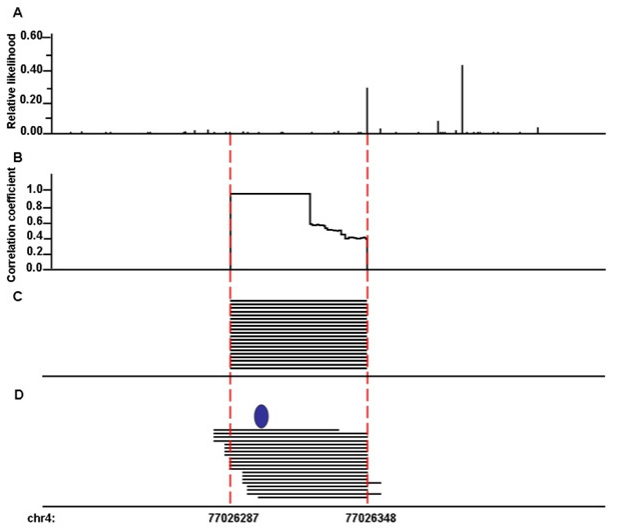
**Genomic region that contains one protein binding site**. A. Relative likelihood of RNase digestion at each genomic locus. B. Similarity between the distribution of experimentally-detected amplicons and simulated amplicons assuming protein binding occurs at the each locus. A higher correlation coefficient implies higher probability of protein binding. C. Distribution of detected RNA fragments. D. Distribution of simulated fragments based on the best prediction locus (marked as blue ellipse).

One enriched region on chr4: 77,026,287-77,026,348 contains 20 identical RNA fragments (Fig. 5C). This is in the second intron of *PPEF2* (Protein phosphatase EF-hand calcium-binding domain 2).

We generated a distribution of RNA fragments based on the likelihood of RNase digestion along the local chromosomal region (with an extension of 70-bp in both 5′- and 3′-ends; Eq. 1 and Fig. 5A). For each potential binding site in the region, we selected 20 virtual RNA fragments that encompass the assumed binding site. The correlation of distribution pattern between experimentally-determined fragments (Fig. 5C) and simulated fragments (Fig. 5D) was calculated (Fig. 5B). Within this enriched region, it is clear that the putative binding sites within the first 36-bp have higher correlation coefficients (all above 96%) with the actual data than did other binding site candidates. Fig. 5D demonstrates the distribution of simulated RNA fragments by assuming protein binds at the location that has the highest correlation coefficient (marked with blue ellipse). Using the same approach on several regions, the resolution of the binding region could be improved by over 70%.

2) Genomic regions that contain two protein binding sites

The proposed approach can also be used to refine the detection of protein binding regions that contain two or more binding sites. Instead of searching for the position of one binding site, as shown in Fig. 3B, positions of two or more putative sites will be optimized to achieve similar pattern distribution between simulated amplicons and experimentally-detected amplicons. This will increase the dimension of searching space.

**Figure 6:**
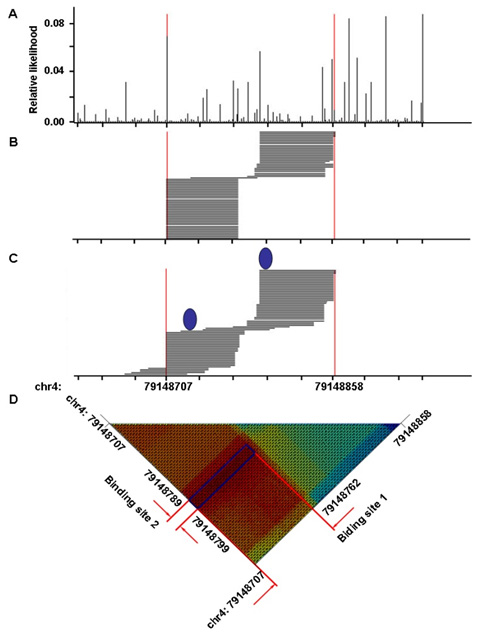
**Genomic region that contains two protein binding site.** A. Relative likelihood of RNase digestion at each genomic locus. B. Distribution of detected RNA fragments. C. Distribution of simulated fragments based on the best prediction loci (marked as blue ellipse). D. Similarity between the distribution of experimentally-detected amplicons and simulated amplicons assuming that protein binding occurs at the each pair of genomic loci. The blue frame indicates the region where highest similarity is observed (dark red).

One example of two-binding-site region, located in the first intron of gene MRPL1 (mitochondrial ribosomal protein L1), is shown in Fig. 6. Within this region, chr4: 79,148,707-79,148,858, we observed 73 overlapped RNA fragments, whose distribution patterns are shown in Fig. 6B. It is clear that the enriched region contains more than one protein binding site. A correlation coefficient matrix was calculated, where each element (*i*, *j*) represents the similarity of the distribution pattern between the experimentally-detected fragments (Fig. 6B) and simulated fragments by hypothetically assuming positions *i* and *j* being the protein binding sites (Fig. 6C). The numbers in this matrix are shown in Fig. 6D, where darker color implies higher correlation coefficient. We observed higher correlation coefficient in one sub-area in the matrix (blue framed region in Fig. 6D), where *i*Ε[79148707,79148762] and *j*Ε[79148789,79148799]. This implies that the two binding sites are probably located in this sub-area.

### Effects of biological and computational variations on binding site prediction

To evaluate the robustness of the proposed approach to various sources of experimental noises from both biological and computational steps, we created a simulated set of virtual amplicons by assuming known protein binding sites and deliberately introducing three different types of noise: antibody non-specificity, inaccurate estimation of sequence specificity of RNase digestion, and *blastn* mapping errors.

a) Antibody non-specificity

Biological noise caused by antibody non-specificity may potentially undermine the ability of the proposed approach to accurately identify protein binding regions. Within the genomic region that is enriched by the detected RNA fragments, antibody non-specificity may cause false detection of RNA fragments that are not really bound by the proteins. These fragments may or may not encompass the real protein binding loci.

We tested the ability of the proposed approach to detect the pre-defined protein binding site while introducing different levels of antibody non-specificity. Within a certain genomic region, we composed a set of *N* virtual fragments (50bp to 70bp) that were generated from two sources:

1. *n* fragments that appeared randomly within the region, with no association with the pre-defined protein binding site and no correlation with 6-bp motif composition of local sequence. Hypothetically, these *n* fragments result from antibody non-specificity.

2. *N-n* fragments that were simulated based on the 6-bp motif composition of local sequence and pre-defined protein binding site. These *N-n* fragments simulate the desired RNA fragments that associate with the studied protein.

**Figure 7:**
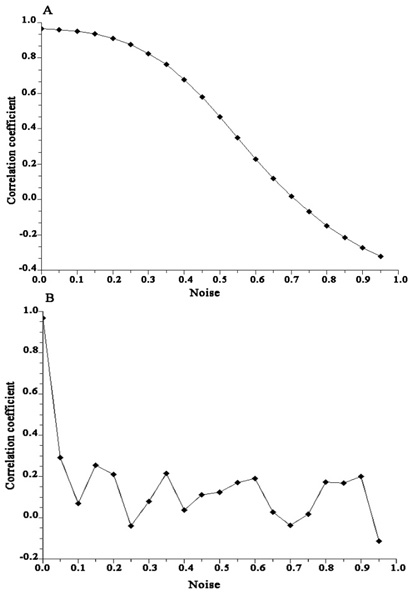
**Effects of computational and biological variations on binding site prediction**. A. Robustness of binding site prediction on antibody non-specificity and *blastn* inaccuracy. B. Robustness of binding site prediction on the inaccurate estimation of sequence specificity of RNase digestion.

Fig. 7A demonstrated the correlation coefficient of the number of RNA fragments that encompass each genomic locus in a comparison between the fragments simulated without noise (procedure 2) and the set combining the simulated and random fragments in different proportions. Not surprisingly, the correlation coefficient decreases with the increase of the proportion of randomly-added RNA fragments (*n/N*). It is clear, however, that the proposed approach retains reasonably good robustness against antibody non-specificity while the proportion of detected RNA fragments contains less than 30% noise, with the correlation coefficient > 80%.

b) Inaccurate estimation of sequence specificity of RNase digestion.

The inaccurate estimation of the sequence specificity of RNase digestion may potentially decrease the accuracy of the model prediction. In this step, we deliberately added measurement noise to the estimated likelihood of RNase digestion on each 6-bp motif and tested the ability of the proposed approach to recover the pre-defined protein binding site. The correlation coefficient of the sequence frequency (number of RNA fragments that encompass each genomic locus) between RNA fragments simulated with and without added noise are shown in Fig. 7B. The accuracy of the model dropped rapidly with the increase of estimation noise of RNase digestion. Therefore, an accurate estimation of the RNase sequence preference is a crucial step in the proposed approach.

c) Noise from BLAST

Mapping the detected fragment back to human transcriptome using *blastn* is not always accurate. This is in part due to the sequencing error in the experimental stage, or due to the *blastn* parameters used. The consequences of BLAST noise on the model prediction are similar to the prediction inaccuracy caused by the antibody non-specificity, i.e. RNA fragments are inaccurately assigned to the studied genomic region. These RNA fragments usually have no association with the real protein binding sites, and have no correlation with the 6-bp motif composition of the studied genomic region. The effect of such noise on the model prediction is evaluated in the Fig. 7A. The model was robust against the noise resulted from BLAST inaccuracy.

## Discussion

In this study, we propose a computational approach to refine the detection of protein binding regions from pyro-sequencing-derived RNA fragments. As an ultra-high-throughput technology, pyro-sequencing allows sequencing of more than 100,000 amplicons in a single experiment [10]. Combined with antibody-based technologies such as chromatin immunoprecipitation (ChIP) or cross-linking immunoprecipitation (CLIP), this technology provides a great opportunity for the researchers to identify *de novo* binding regions of DNA/RNA binding proteins in a genome-wide scale.

Using pyro-CLIP experiment as an example, we present a computational framework to increase the detection resolution within an RNA-fragment-enriched region by using (1) the distribution patterns of the detected amplicons (Fig. 1B, 2A, 5C and 6B), and (2) the sequence specificity of RNase digestion along the local genomic sequences of the enriched region.

Multiple RNA fragments can be detected in each enriched region. Intuitively, protein binding region can be localized by finding the common genomic regions that are encompassed by all the local fragments. This is based on the assumption that only those RNA fragments that contain the protein binding site can be immunoprecipated by the antibody, and therefore can be detected by the system. Localization is compromised by the sequence selectivity of RNase digestion. Therefore, the distribution pattern of the RNA fragments contains less information than the one that would result from random sequence cutting. Incorporating the sequence preference of the RNase digestion into the model can partially recover the information. In conclusion, the proposed approach demonstrated great potential in refining the detection of protein binding region using pyrosequencing-derived RNA fragments.

## Competing interests

The authors declare that they have no competing interests.

## Authors' contributions

YL and JRS contributed to the design of the study. JRS and XW performed the CLIP and pyrosequencing assays. XW and YL designed and performed the bioinformatics analysis and drafted the manuscript. CS, LL, SDM, HJE, and JRS participated in coordination, discussions related to result interpretation and revision of the manuscript. All the authors read and approved the final manuscript.
